# In Situ-Prepared Nickel Oxide Electrodes for Electrochemical Detection of Nitrite via Catalytic Reduction Mechanism

**DOI:** 10.3390/s26102932

**Published:** 2026-05-07

**Authors:** Yihao Geng, Huicong Zhou, Siyuan Lu, Guanyue Wang, Xing Zhao, Hui Suo, Chun Zhao

**Affiliations:** State Key Laboratory of Integrated Optoelectronics, JLU Region College of Electronic Science and Engineering, Jilin University, Changchun 130012, China; gengyh24@mails.jlu.edu.cn (Y.G.); zhouhc25@mails.jlu.edu.cn (H.Z.); lusy1223@mails.jlu.edu.cn (S.L.); 15603105787@163.com (G.W.); zhaoxing24@mails.jlu.edu.cn (X.Z.); suohui@jlu.edu.cn (H.S.)

**Keywords:** electrochemical detection, nitrite, low reduction potential, NiO nanoflowers

## Abstract

In electrochemical nitrite detection, the strong oxidizing nature of nitrite often leads to high detection potentials, posing a significant challenge. To address this issue, this study successfully fabricated a nickel oxide/carbon cloth (NiO/CC) electrode using a one-step electrodeposition method followed by calcination. Taking advantage of the excellent electrocatalytic reduction properties of nickel oxide—particularly the surface oxygen vacancies that serve as active sites for efficient nitrite ion adsorption and promote the hydrogenation of the key intermediate (*NO)—the reaction energy barrier is substantially reduced. As a result, the NiO/CC electrode enables high-sensitivity nitrite detection at a low potential. Electrochemical evaluations reveal that the NiO/CC sensor performs excellently at −0.15 V (vs. Hg/HgO), featuring a linear detection range of 10–500 μM, a low detection limit of 0.091 μM (S/N = 3), and a high sensitivity of 2910 μA·mM^−1^·cm^−2^. These results highlight the promise of a catalytic reduction-based strategy for lowering detection potentials and provide a crucial foundation for the rational design of high-performance electrochemical sensing interfaces.

## 1. Introduction

Nitrite (NO_2_^−^) is widely present in the environment and food, making its detection critically important for ecological safety and public health. As a key intermediate in the nitrogen cycle, nitrite plays an indispensable role in microbial-mediated nitrification and denitrification processes [1]. Additionally, owing to its excellent color-protecting and antibacterial effects, it is commonly used as an additive in meat products. However, excessive intake of nitrite can cause methemoglobinemia and may react with amines in gastric acid to form highly carcinogenic nitrosamines [2]. Furthermore, excess nitrite in the environment can exacerbate water eutrophication and threaten aquatic ecosystems [3]. Given this dual nature—being both ecologically necessary and potentially hazardous—the development of highly sensitive and selective nitrite detection methods has become a research hotspot in analytical chemistry. Compared with the limitations of traditional chromatography and spectroscopy techniques, such as expensive instrumentation and cumbersome pretreatment, electrochemical sensing technology offers advantages including rapid response, simple operation, and ease of miniaturization, thereby demonstrating broad application prospects for on-site rapid detection [4].

Current electrochemical nitrite sensors mainly rely on high-potential oxidation strategies. However, the relatively high working potential often induces side reactions, thereby compromising detection accuracy [5]. Given that nitrite possesses strong reducibility and a relatively low reduction potential, strategies based on reduction current offer a highly promising alternative approach, effectively avoiding interference under high potential and improving detection accuracy and stability [6]. For example, Zhao et al. fabricated copper–tin alloy nanosheet/carbon paper electrodes (CuSn/CP) via one-step electrodeposition, achieving excellent sensing performance at a working potential of −0.08 V (vs. Hg/HgO), with a sensitivity as high as 4920 μA·mM^−1^·cm^−2^, a detection limit as low as 0.089 μM, and outstanding anti-interference capability [7]. Similarly, Amini et al. modified a glassy carbon electrode with nanocomposite materials and demonstrated significant electrocatalytic activity for nitrite reduction at a low potential of 0.12 V, achieving a detection limit of 7 μM and a sensitivity of 0.0007 μA/μM [8]. These studies collectively confirm the feasibility and practical value of constructing low-potential electrochemical sensors based on the catalytic reduction mechanism of nitrite, providing new research directions for the development of high-sensitivity nitrite detection technologies [9].

Nickel oxide (NiO) exhibits outstanding performance in the catalytic reduction of nitrite due to its abundant surface oxygen vacancies and unique electronic structure. The strong interaction between nickel active sites and nitrite radicals optimizes the reaction product adsorption configuration and effectively promotes the reduction reaction by lowering the energy barrier for the N=O bond breakage [10]. The redox couple of Ni^2+^/Ni^3+^ on the NiO surface provides a convenient channel for electron transfer, endowing the material with high catalytic activity, rapid response, and a lower reduction overpotential. Its inherent oxygen vacancies can serve as specific adsorption sites, selectively enriching nitrite radicals and achieving efficient catalytic conversion within a narrow potential window, thereby effectively suppressing side reactions and background interference [11].

Furthermore, the selection of the sensitive electrode substrate is critically important for constructing high-performance nitrite sensors [12]. Carbon cloth, as an excellent three-dimensional conductive carbon material, not only possesses good flexibility and a large specific surface area but also promotes rapid diffusion and penetration of the electrolyte [13]. Therefore, in situ growth of nickel oxide nanostructures on the carbon cloth substrate provides an effective strategy for preparing a binder-free electrochemical nitrite sensor. The synergistic combination of nickel oxide and carbon cloth fully leverages their respective advantages: the three-dimensional conductive network of carbon cloth provides fast channels for electron transfer, effectively compensating for the relatively insufficient conductivity of the nickel oxide semiconductor material [14]; simultaneously, the in situ growth of nickel oxide nanostructures on the surface of carbon cloth fibers not only increases the exposed area of active sites but also avoids the coating and blocking of active sites by binders that occur in conventional coating methods [15]. The combination of the excellent mechanical flexibility of carbon cloth and the stable crystal structure of nickel oxide endows the electrode with outstanding strain resistance and long-term cycling stability. Consequently, the nickel oxide/carbon cloth composite electrode holds great promise for nitrite detection with high sensitivity, low detection potential, high selectivity, and excellent stability [16,17,18,19,20].

Therefore, this study successfully constructed a self-supporting nickel oxide nanoflower/carbon cloth (NiO/CC) sensing electrode via electrodeposition combined with calcination, achieving low-potential and high-selectivity detection of nitrite. The research results demonstrate that the NiO/CC electrode exhibits excellent comprehensive performance in nitrite sensing, including high sensitivity, a low detection limit, a low working potential, outstanding anti-interference capability, and superior stability. These advantageous characteristics primarily arise from the synergistic effect between the abundant oxygen vacancies on the nickel oxide surface and the three-dimensional conductive network of the carbon cloth: the oxygen vacancies effectively adsorb nitrite ions and promote the hydrogenation conversion of key intermediates, thereby lowering the reaction energy barrier and improving catalytic efficiency. Additionally, the intimate interfacial contact between nickel oxide and carbon cloth provides fast channels for electron transfer, further enhancing the electrocatalytic performance. This mechanism has been preliminarily verified through relevant electrochemical characterizations. More importantly, the self-supporting NiO/CC electrode demonstrates good practicality and reliability in real-sample detection. This study elucidates the mechanism of nickel oxide in the catalytic reduction process of nitrite and its excellent tolerance to inorganic nitrogen interfering species, providing an effective solution based on a catalytic reduction strategy for stable electrochemical detection of nitrite.

## 2. Materials and Methods

### 2.1. Chemical Reagent

All chemicals are of analytical grade and can be used without further purification. Nickel chloride hexahydrate (NiCl_2_·6H_2_O), sodium nitrite (NaNO_2_), potassium chloride (KCl), potassium ferrocyanide (K_3_[Fe(CN)_6_]), and calcium chloride (CaCl_2_) were purchased from the Beijing Chemical Factory Co (Beijing, China). Ammonium chloride (NH_4_Cl), potassium carbonate (K_2_CO_3_), magnesium sulfate heptahydrate (MgSO_4_·7H_2_O), sodium nitrate (NaNO_3_), ferric chloride hexahydrate (FeCl_3_-6H_2_O), and potassium hydroxide (KOH) were supplied by China Shanghai Sinopharm Chemical Reagent Co (Shanghai, China). Hydrochloric acid (HCl), glucose (C_6_H_12_O_6_), and urea (CO(NH_2_)_2_) were purchased from Tianjin Yongda Chemical Reagent Co., Ltd. (Tianjin, China). Carbon cloth (CC) was purchased from Carbon Enger Technology Co., Ltd. (Taichung City, China).

### 2.2. Preparation of Electrodes

#### 2.2.1. Pre-Treatment of Carbon Cloth

The carbon cloth (CC) was cut into small rectangular pieces (2 × 1 cm^2^) and then sequentially sonicated in toluene, acetone, ethanol, 0.5 M hydrochloric acid, and deionized water for 10 min each. Afterward, the carbon cloth was rinsed with deionized water until neutral pH was achieved and stored in deionized water for future use.

#### 2.2.2. Preparation of Sensitive Electrodes

This sensing electrode is fabricated through a two-step process combining constant potential electrodeposition and subsequent calcination. Firstly, different concentrations (5, 10, 30, 50 mM) of hexahydrate nickel chloride (NiCl_2_·6H_2_O) are dissolved in deionized water, and 0.1 M potassium chloride (KCl) is added as the supporting electrolyte to prepare a uniform precursor solution. Carbon cloth that has been pre-treated is used as the working electrode. A constant potential of −1.0 V (vs. Ag/AgCl) is applied in a three-electrode system for deposition. The deposition time is set at 300, 600, 900, and 1200 s, respectively, to optimize the thickness and morphology of the deposited layer. After deposition, the electrode is thoroughly rinsed with deionized water to remove the residual electrolyte on the surface and then dried at 60 °C for 1 h for subsequent processing.

To further enhance the electrochemical performance of the electrode and optimize its surface activity, the dried electrodes were subjected to calcination treatment in an air atmosphere. The calcination temperatures were set at 100, 200, 300, and 400 °C, and the holding times were 1, 2, 3, and 4 h, respectively. This was done to systematically investigate the influence of thermal treatment conditions on the electrocatalytic performance of nickel oxide. After optimization and screening, the final product was named NiO/CC electrode and was used for subsequent electrochemical characterization and nitrite detection studies.

### 2.3. Electrochemical Measurements and Characterizations

The surface morphology and microstructure of the prepared electrodes were investigated using field emission scanning electron microscopy (SEM, JEOL-JEM-6700F, JEOL, Ltd., Tokyo, Japan). The crystal structure of the prepared electrode was characterized using X-ray diffraction spectroscopy (XRD, D8 ADVANCE, Cu Kα source (λ = 1.54 Å), Bruker AXS, Karlsruhe, Germany). The elemental composition and chemical valence states of the prepared electrodes were analyzed using X-ray photoelectron spectroscopy (XPS, ESCALAB-250, Thermo Fisher Scientific, Waltham, MA, USA).

Electrochemical susceptibility testing of the sensitive electrodes was conducted using an electrochemical workstation (CHI760D, Shanghai Chenhua Instrument Co., Ltd., Shanghai, China) with a three-electrode system: NiO/CC as the working electrode, Hg/HgO as the reference electrode, and a 1.5 cm × 1.5 cm Pt sheet as the counter electrode. Cyclic voltammetry (CV) tests were performed in a 0.1 M KOH electrolyte, with a potential range of −0.4 V to +0.5 V and a scan rate of 50 mV s^−1^. Additionally, an i-t test was conducted under stirring at a working potential of −0.15 V vs. Hg/HgO in the same electrolyte. To analyze the electrochemical activity of the electrodes, further tests were performed in a 5 mM potassium ferricyanide solution using the same three-electrode setup, but with an Ag/AgCl reference electrode. These tests included electrochemical specific surface area measurement and electrochemical impedance spectroscopy (EIS). The EIS test was conducted with an amplitude of 5 mV and a frequency range of 10^−2^ kHz to 10^−5^ kHz. The electrochemical specific surface area was determined by CV within a potential range of −0.2 V to +0.6 V, with scanning rates varying from 20 to 160 mV s^−1^.

### 2.4. Preparation of Real Samples

Pickled vegetable samples were purchased from a local farmers’ market. The pretreatment procedure was as follows: 50 g of pickled vegetables were homogenized for 5 min, and the homogenate was rinsed with deionized water and diluted to 1000 mL. Then, 50 mL of the above mixture was centrifuged at 5000 rpm for 20 min. The supernatant was mixed with 0.1 M NaOH at a volume ratio of 1:9 (i.e., 5 mL of supernatant and 45 mL of NaOH), followed by stirring for 30 min to form a stable test electrolyte for subsequent electrochemical detection. For drinking water samples, the water sample was directly mixed with 0.1 M NaOH at a volume ratio of 1:9 (5 mL of water sample and 45 mL of NaOH) and stirred for 30 min before testing.

## 3. Results and Discussion

### 3.1. Characterization of Sensitive Electrodes

**Figure 1:**
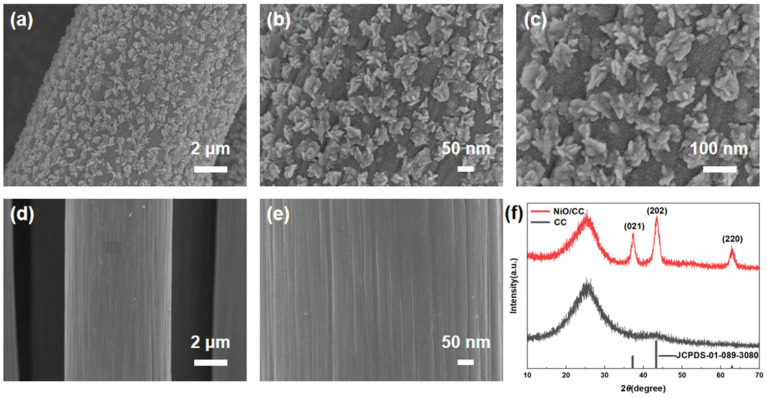
(**a**–**c**) SEM images of NiO/CC at different magnifications, showing the uniform growth of NiO nanoflowers on carbon cloth fibers; (**d**,**e**) SEM images of bare carbon cloth (CC) with smooth fiber surfaces; (**f**) XRD patterns of NiO/CC and bare CC. The peaks labeled with (021), (202), and (220) correspond to the crystal plane indices of cubic NiO (JCPDS-01-089-3080).

The microscopic morphology of the nickel oxide/carbon cloth (NiO/CC) sensing electrode and the bare carbon cloth was characterized using a scanning electron microscope (SEM), with the results shown in Figure 1a–e. Figure 1d,e present the SEM images of the bare carbon cloth, revealing that it is composed of numerous interwoven carbon fibers with smooth surfaces and an average diameter of approximately 2 μm. Figure 1a–c display the SEM images of the NiO/CC sensing electrode at different magnifications. From the low-magnification image (Figure 1b), it can be observed that nickel oxide nanoparticles grow uniformly and densely on the surface of each carbon fiber. Further magnification (Figure 1c) reveals that these nickel oxides exhibit a three-dimensional nanoflower-like structure, consisting of a large number of ultrathin nanopetals. This unique nanoflower morphology significantly increases the effective specific surface area of the sensing electrode, providing abundant active sites for nitrite adsorption and catalytic reaction, promoting sufficient contact and mass transfer between the analyte and the electrode surface, and thereby substantially enhancing the detection sensitivity of the sensor for nitrite [21,22,23].

Furthermore, the crystal structures of the nickel oxide/carbon cloth (NiO/CC) sensitive electrode and the blank carbon cloth were analyzed using X-ray diffraction spectroscopy (XRD). The results are shown in Figure 1f. In contrast, the XRD pattern of the NiO/CC sensitive electrode not only retains the diffraction peaks of the carbon cloth but also shows new peaks at 37.2°, 43.3°, and 62.9°, which correspond to the (021), (202), and (220) crystal planes of cubic crystal system nickel oxide (NiO) (JCPDS-01-089-3080). It is noteworthy that no characteristic diffraction peaks of metallic nickel, nickel hydroxide, or other impurities were detected in the spectrum, indicating that the prepared nickel oxide has high phase purity and good crystallinity. This result confirms that the method of electrode deposition combined with calcination successfully constructed a pure nickel oxide crystal structure on the surface of the carbon cloth, providing a structural basis for its excellent electrocatalytic performance [7,24,25,26].

**Figure 2:**
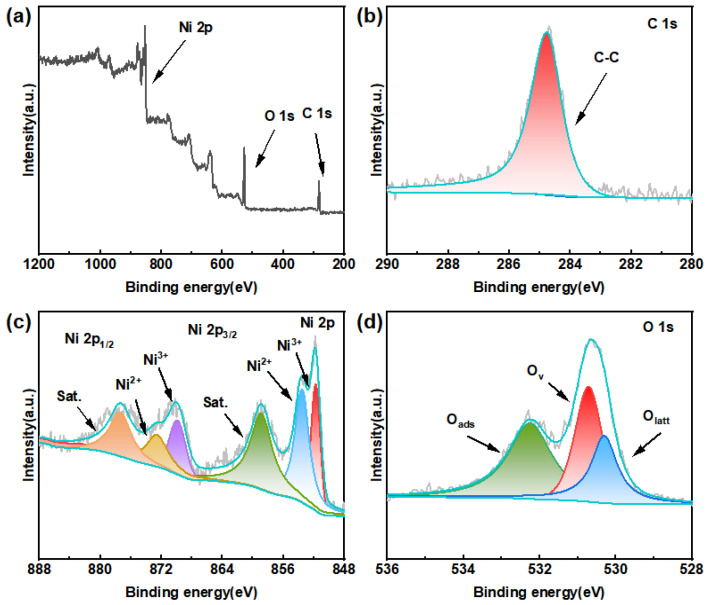
XPS spectra of NiO/CC electrodes: (**a**) measured spectrum, (**b**) C1, (**c**) Ni 2p, (**d**) O 1s.

The elemental composition and chemical valence of the nickel oxide/carbon cloth (NiO/CC) sensitive electrode were analyzed in depth by X-ray photoelectron spectroscopy (XPS). The results are shown in Figure 2a–d. The full-spectrum scan in Figure 2a revealed characteristic peaks of three elements, namely Ni, O, and C, on the electrode surface. No other impurity elements were detected, indicating that the prepared NiO/CC electrode has high chemical purity. Among them, the C element originated from the carbon cloth substrate, while the Ni and O elements belonged to the surface-loaded nickel oxide layer.

Figure 2b shows the high-resolution energy spectrum of C 1s. Only a symmetrical peak at 284.8 eV was observed, corresponding to the C-C bond, confirming the graphite-like structure of the carbon cloth substrate, and no interference from other carbon-containing functional groups was detected [27,28,29].

Figure 2c presents the high-resolution XPS energy spectrum of Ni 2p. Through peak fitting, it can be observed that the Ni 2p spectrum shows two pairs of spin–orbit double peaks and their accompanying satellite peaks (denoted as “Sat.”). Among them, the double peaks with binding energies at 853.7 eV and 872.7 eV correspond to Ni 2p3/2 and Ni 2p1/2, respectively, belonging to the oxidation state of Ni^2+^; while the double peaks at 851.6 eV and 870.1 eV belong to the oxidation state of Ni^3+^. This result indicates that the prepared oxidized nickel surface simultaneously contains Ni^2+^ and Ni^3+^, and the existence of this mixed valence state is conducive to the formation of abundant oxygen vacancy defects and promotes the electron transfer between the electrode surface and the nitrite, thereby enhancing the electrocatalytic activity [30,31].

Figure 2d shows the high-resolution XPS spectrum of O 1s. After peak separation and fitting, it can be decomposed into three characteristic peaks. The peak at 530.7 eV belongs to lattice oxygen (Olatt), corresponding to O^2−^ in the NiO crystal structure; the peak at 530.2 eV belongs to oxygen vacancies or defective oxygen (Ov); and the peak at 532.3 eV belongs to surface adsorbed oxygen (Oads), such as surface hydroxyl groups (-OH) or adsorbed water molecules. The appearance of the oxygen vacancy characteristic peak further confirms the presence of abundant defect sites on the surface of oxidized nickel, which can act as active centers to effectively adsorb nitrite ions and promote the progress of the catalytic reduction reaction.

The above XPS analysis results are consistent with the XRD characterization, further confirming that by combining electrodeposition with calcination, a pure nickel oxide sensitive layer rich in oxygen vacancy defects and with mixed valence characteristics was successfully constructed on the surface of the carbon cloth. This provides a sufficient surface chemical basis for its excellent low-potential nitrite catalytic reduction performance.

### 3.2. Electrochemical Behaviors of NiO/CC

**Figure 3:**
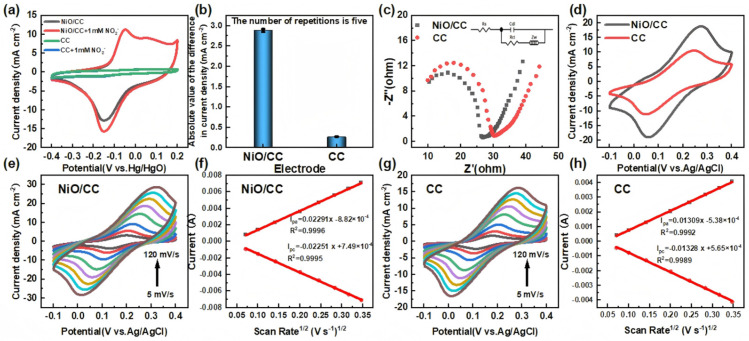
(**a**) Cyclic voltammograms of NiO/CC and CC electrodes before and after adding NO_2_^−^ in 0.1 M KOH electrolyte; (**b**) Histogram of the absolute values of the difference in corresponding reduction peak current densities; (**c**) Electrochemical impedance spectra (EIS) Nyquist plots of NiO/CC and bare CC electrodes in 0.1 M KCl solution containing 5.0 mM [Fe(CN)_6_]^3−^/^4−^; (**d**) Cyclic voltammograms of the two electrodes under the same conditions; (**e**) Cyclic voltammograms of NiO/CC electrode and (**g**) bare CC electrode at different scan rates (20 to 200 mV·s^−1^) (electrolyte conditions are the same as before); (**f**,**h**) Linear fitting relationships between peak oxidation current of NiO/CC and square root of scan rate (v^1^/^2^) of NiO/CC and bare CC electrodes.

By comparing the cyclic voltammetry response curves before and after the addition of nitrite (Figure 3a,b), it was found that the reduction peak current significantly increased after the addition of nitrite, and the response degree of different modified electrodes was significantly different: The NiO/CC electrode showed the highest reduction peak current and the lowest initial reduction potential, while the response of the bare CC electrode was almost negligible, indicating that the NiO modification layer played a crucial role in the electrocatalytic process. Further analysis of the absolute value of the difference in reduction peak current density (Figure 3b) revealed that the possible catalytic mechanism of NiO includes: Its surface with high density of active sites is conducive to the adsorption of nitrite ions, and the redox cycle of Ni (Ni^2+^/Ni^3+^) as an electron transfer medium accelerates the conversion of nitrite to nitrogen gas. In conclusion, Figure 3a,b not only verified the electrocatalytic reduction behavior of nitrite by the NiO/CC electrode but also provided an electrochemical basis for the subsequent performance evaluation of the sensor.

The electron transfer kinetics and interfacial electrochemical characteristics of the NiO/CC sensitive electrode and the bare carbon cloth (CC) electrode were comparatively investigated, with the results presented in Figure 3c,d. Figure 3d shows the cyclic voltammograms of the two electrodes in a 0.1 M KCl solution containing 5 mM [Fe(CN)_6_]^3−^/^4−^. Both electrodes exhibit a pair of typical reversible redox peaks, indicating that the electrode processes are diffusion-controlled. Notably, the oxidation and reduction peak current densities of the NiO/CC electrode are approximately twice those of the bare CC electrode, which directly demonstrates its larger electrochemical active surface area and superior electron transfer capability. This enhancement is primarily attributed to the successful construction of oxygen vacancy-rich nickel oxide nanoflower structures on the carbon cloth surface: on one hand, the three-dimensional nanoflower morphology greatly increases the specific surface area of the electrode, providing more active sites for redox probes; on the other hand, the abundant oxygen vacancy defects on the nickel oxide surface effectively modulate the electronic structure at the electrode/electrolyte interface, accelerating interfacial charge transfer kinetics and thereby significantly enhancing the current response of the redox reaction.

**Figure 4:**
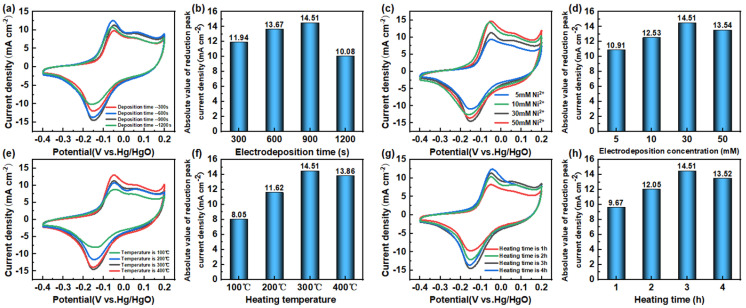
Cyclic voltammograms of NiO/CC sensitive electrodes prepared under different deposition times, the curve graph after adding 0.6 mM nitrite to 0.1 M KOH electrolyte (**a**), and the bar graph showing the decrease in the absolute peak current of the CV curve (**b**); Cyclic voltammograms of NiO/CC sensitive electrodes prepared under different deposition concentrations, the curve graph after adding 0.6 mM nitrite to 0.1 M KOH electrolyte (**c**), and the bar graph showing the decrease in the absolute peak current of the CV curve (**d**); Cyclic voltammograms of NiO/CC sensitive electrodes prepared under different calcination temperatures, the curve graph after adding 0.6 mM nitrite to 0.1 M KOH electrolyte (**e**), and the bar graph showing the decrease in the absolute peak current of the CV curve (**f**); Cyclic voltammograms of NiO/CC sensitive electrodes prepared under different calcination times, the curve graph after adding 0.6 mM nitrite to 0.1 M KOH electrolyte (**g**), and the bar graph showing the decrease in the absolute peak current of the CV curve (**h**).

Figure 3c presents the Nyquist plots of the NiO/CC electrode and the blank carbon cloth electrode, with the corresponding equivalent circuit models inserted. The Nyquist plots are typically composed of a semi-circle in the high-frequency region and a straight line in the low-frequency region, corresponding to the charge transfer process and the diffusion process, respectively. The size of the charge transfer resistance (Rct) can be reflected by the diameter of the semi-circle—the smaller the diameter, the lower the Rct, which is more conducive to the progress of electrochemical reaction kinetics. It can be clearly seen from Figure 4b that the diameter of the semi-circle in the high-frequency region of the NiO/CC sensitive electrode is significantly smaller than that of the blank carbon cloth electrode, indicating that the in situ growth of nickel oxide nanowedges on the carbon cloth surface effectively reduces the interface charge transfer resistance. Further, through equivalent circuit fitting, the Rct values of the NiO/CC electrode and the blank carbon cloth electrode are 24.77 Ω and 31.15 Ω, respectively. This result fully confirms that the introduction of nickel oxide significantly improves the electron transmission ability of the electrode interface, reduces the charge transfer energy barrier, and effectively accelerates the electrochemical reaction process.

It should be noted that the above EIS measurements were performed in the Fe^2+^/Fe^3+^ redox system, which mainly reflects the charge transfer resistance and electrochemically active area of the electrode. Since the electrochemical reduction of nitrite may involve inner-sphere electron transfer, adsorption processes, or chemical coupling steps—mechanisms that differ from the outer-sphere electron transfer of the Fe^2+^/Fe^3+^ probe—the EIS results in this system do not necessarily fully represent the electrocatalytic behavior of nitrite on the NiO/CC electrode. Therefore, the EIS data are used here primarily to evaluate electrode conductivity and effective area, not as direct evidence of nitrite electrocatalysis.

To further evaluate the electrochemical active specific surface area (ECSA) of the two electrodes, cyclic voltammetry tests were conducted on the NiO/CC sensitive electrode and the blank carbon cloth electrode at different scan rates in a 0.1 M KCl solution containing 5 mM [Fe(CN)_6_]^3−^/^4−^. The scan rates were set at 5, 10, 20, 40, 60, 80, 100, and 120 mV/s, and the results are shown in Figure 4e–h. The experimental results indicate that as the scan rate increases, the oxidation peak current (Ipa) of the NiO/CC electrode gradually increases, and it shows a good linear relationship with the square root of the scan rate (v^1^/^2^), indicating that the electrode process is controlled by diffusion. Based on the Langdon–Schweizer equation (as shown in Formula (1)), it was calculated that the electrochemical active specific surface area of the NiO/CC electrode is significantly higher than that of the blank carbon cloth electrode. This result further confirms that the nickel oxide flower structure effectively increases the active reaction area of the electrode, thereby providing a strong electrochemical basis for its excellent nitrite sensing performance [19,20,21].

(1) $$I_{\mathrm{pa}}=\left(2.69\times 10^{5}\right)n^{\frac{3}{2}}ACD^{\frac{1}{2}}\nu^{\frac{1}{2}}$$

In Equation (1), Ipa, n, A, C, D, and ν represent the oxidation peak current (A), the number of electrons transferred (*n* = 3), the electrochemical active surface area of the electrode (cm^2^), the concentration of the probe molecules (5 × 10^−6^ mol·cm^−3^), the diffusion coefficient (7.60 × 10^−6^ cm^2^·s^−1^), and the scan rate (V·s^−1^), respectively. Based on this equation, the ECSA values for the NiO/CC sensitive electrode and the bare CC electrode were calculated to be 11.48 cm^2^ and 6.56 cm^2^, respectively. These results indicate that the introduction of metallic copper nanosheets onto the carbon paper surface effectively enhances the overall performance of the electrode, as evidenced by the reduced charge transfer resistance, improved conductivity, increased electrochemical active surface area, greater number of electroactive sites, faster electron transfer rate, and enhanced electrocatalytic activity. These synergistic improvements significantly promote the electrocatalytic reduction of nitrite, thereby effectively enhancing the detection performance of the sensitive electrode.

### 3.3. Sensitive Determination of Nitrite

The preparation conditions of the nickel oxide/carbon cloth (NiO/CC) sensitive electrode were systematically optimized through electro-deposition and subsequent calcination processes to determine the key preparation parameters. The results are shown in Figure 4. During the electro-deposition process, the effects of precursor concentration and deposition time on the electrode performance were investigated. The experiments indicated that when the concentration of NiCl_2_·6H_2_O was 30 mM and the deposition time was 900 s, the loading amount of nickel oxide nanowedges on the carbon cloth surface was the most suitable. A low concentration (such as 5 and 10 mM) resulted in insufficient active material loading and limited catalytic sites; while a high concentration (such as 50 mM) was prone to cause nanostructure aggregation, thereby reducing the effective specific surface area. Similarly, a short deposition time (300 s) could not form a uniform nanowedge structure, while a long time (1200 s) might lead to an overly thick active layer, hindering electron transmission.

After the electro-deposition process is completed, the obtained precursor electrodes are further subjected to calcination treatment to optimize the crystal structure and surface defects of the nickel oxide. The effects of calcination temperature (100, 200, 300, 400 °C) and holding time (1, 2, 3, 4 h) were investigated. The results showed that the electrode prepared by calcination at 300 °C for 3 h exhibited the best electrocatalytic performance. An appropriate calcination temperature helps the precursor fully convert into cubic crystal system nickel oxide and simultaneously induces the generation of abundant oxygen vacancy defects, which can serve as active centers for the adsorption and catalysis of nitrite. Low temperatures (200 °C) may lead to incomplete conversion or insufficient crystallinity, resulting in insufficient exposure of active sites; while high temperatures (400 °C and above) may cause a reduction in oxygen vacancies, sintering collapse of the nanostructure, and thus reducing the catalytic activity. Short holding time (1 h) also leads to incomplete conversion, while long holding time (4 h) may cause excessive grain growth or defect annihilation.

The optimal preparation conditions after comprehensive optimization are as follows: NiCl_2_·6H_2_O concentration of 30 mM, deposition time of 900 s, calcination temperature of 300 °C, and holding time of 3 h. The NiO/CC sensitive electrode prepared under these optimized conditions was selected for the subsequent electrochemical performance evaluation and nitrite detection research.

**Figure 5:**
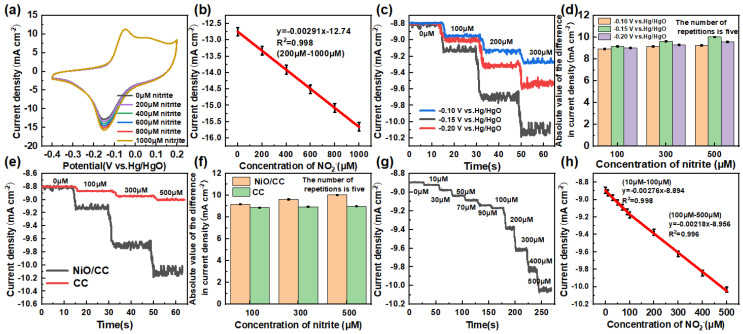
(**a**) CV curves of the NiO/CC electrode in 0.1 M KOH with varying nitrite concentrations; (**b**) Linear fit of reduction peak current vs. nitrite concentration (200–1000 μM); (**c**) Chronoamperometric response to stepwise nitrite addition at different potentials; (**d**) Corresponding current density bar chart; (**e**) Response of different electrodes to nitrite at −0.15 V vs. Hg/HgO; (**f**) Corresponding current density comparison; (**g**) Stirred chronoamperometric response at −0.15 V vs. Hg/HgO. Note that the concentration steps are not uniform; the actual addition sequence and time intervals are shown in the figure. (**h**) Linear calibration of current vs. nitrite concentration for the low-concentration range (10–100 μM) and high-concentration range (100–1000 μM), respectively.

The performance of the nickel oxide/carbon cloth (NiO/CC) sensitive electrode for detecting nitrite was evaluated using cyclic voltammetry. The results are shown in Figure 5a,b. In a 0.1 M KOH electrolyte, as the concentration of nitrite gradually increased, the cyclic voltammogram of the NiO/CC electrode (Figure 5a) exhibited a regular increase in the reduction peak current response. This phenomenon indicates that nitrite ions effectively diffuse to the electrode surface and undergo reduction reactions under the catalytic action of nickel oxide, promoting interface electron transfer. The excellent sensing performance of the above-mentioned electrode is mainly attributed to the unique nanoflower structure of NiO/CC, which provides abundant active sites, a large electrochemical active surface area, and efficient electron transfer capability.

Figure 5b shows the linear relationship between the reduction peak current density of the NiO/CC sensitive electrode and the nitrite concentration. Within the concentration range of 10 to 500 μM, the current response shows a good linear relationship with the nitrite concentration, with the linear regression equation being Ipc (mA·cm^−2^) = −0.00291c (μM) − 12.74, and the correlation coefficient R^2^ = 0.998, indicating excellent linear correlation within this concentration range. Based on the linear slope, the sensor’s sensitivity is as high as 2910 μA·mM^−1^·cm^−2^, which is significantly higher than many reported nitrite electrochemical sensors, fully demonstrating the excellent catalytic activity of the NiO/CC electrode. Based on the signal-to-noise ratio (S/N = 3), the detection limit (LOD) of the sensor for nitrite is as low as 0.091 μM, much lower than the maximum allowable content of nitrite in drinking water recommended by the World Health Organization (WHO) (65.2 μM), fully meeting the detection requirements for trace nitrite in actual samples. The low detection limit is mainly attributed to the abundant active sites provided by the nickel oxide nanoclover structure, the effective enrichment of nitrite by the oxygen vacancy defects, and the excellent three-dimensional conductive network of the carbon cloth substrate that endows it with rapid electron transfer ability [32,33].

In order to achieve highly sensitive detection of low-concentration nitrite, a systematic study was conducted using the time-amperometric method by continuously adding nitrite at different working potentials. The results are shown in Figure 5c,d. The experiment investigated the current response behavior of the nickel oxide/carbon cloth (NiO/CC) electrode to nitrite at different potentials. The results indicated that at the working potential of −0.15 V (vs. Hg/HgO), the NiO/CC electrode exhibited the highest current response value and the clearest concentration gradient resolution ability. Therefore, this potential was determined as the optimal working potential for subsequent detection. This potential was significantly lower than the detection potential required by traditional oxidative sensors, effectively avoiding the influence of possible side reactions and interfering substances at high potentials.

Under the optimized −0.15 V potential, the current response of the NiO/CC electrode to the continuous addition of nitrite was further compared with that of the blank carbon cloth electrode (Figure 5e,f). The results showed that the current response amplitude of the NiO/CC electrode was significantly higher than that of the blank carbon cloth electrode, and the response time was shorter, reaching the steady-state current faster. This excellent performance was mainly attributed to the following aspects: Firstly, the abundant oxygen vacancy defects on the surface of nickel oxide can act as active centers to effectively adsorb nitrite ions, reducing the reaction energy barrier; Secondly, the nanoflower structure of nickel oxide provides a huge specific surface area, increasing the number of exposed active sites; Moreover, the presence of mixed valence states of Ni^2+^/Ni^3+^ in nickel oxide promotes interface electron transfer, accelerating the kinetic process of nitrite reduction reaction. The synergistic effect of these factors enables the NiO/CC electrode to exhibit excellent catalytic activity and sensitive concentration response ability under low potential conditions.

The time course amperometric tests on the nitrite concentration gradient on the NiO/CC electrode (Figure 5g,h) indicated that the current reached a stable state within 1.6 s after nitrite injection, demonstrating the rapid material diffusion and electron transfer kinetics characteristics at the electrode interface. The current response showed a highly linear relationship with the nitrite concentration in the range of 10 to 100 μM, with the linear regression equation being Ipc = −0.00276c − 8.894 (R^2^ = 0.998); in the wide concentration range of 100 to 1000 μM, it still maintained a good linear relationship, with the equation being Ipc = −0.00218c − 8.956 (R^2^ = 0.996). Based on the above results, the sensitivity of the NiO/CC electrode for nitrite detection was calculated to be as high as 2760 μA·mM^−1^·cm^−2^, and the detection limit was as low as 0.091 μM (S/N = 3), which was much lower than the World Health Organization (WHO) recommended standard threshold for nitrite in drinking water (65.2 μM). At a low working potential of −0.15 V (vs. Hg/HgO), this electrode successfully achieved the synergistic optimization of wide linear range and high sensitivity, fully demonstrating its unique advantages in low potential detection. Compared with the electrochemical sensors reported in existing literature (Table 1), the NiO/CC electrode exhibited significant competitive advantages in key performance indicators such as detection potential, linear range, detection limit, and sensitivity, further confirming its broad application prospects in quantitative nitrite detection [34].

### 3.4. Anti-Interference, Stability and Reproducibility of NiO/CC

**Figure 6:**
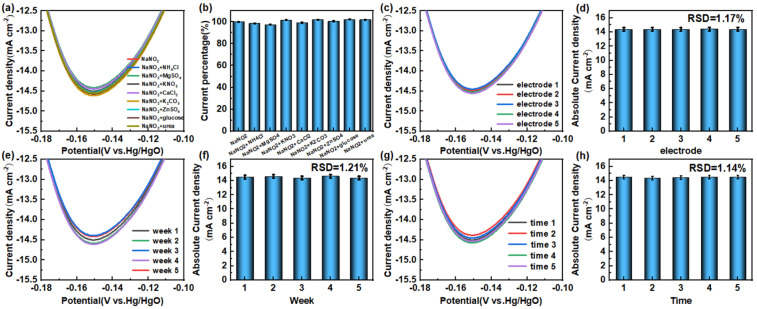
(**a**) Local magnification of the cyclic voltammogram of the NiO/CC sensitive electrode in a 0.1 M KOH electrolyte containing 1 mM nitrite; (**b**) Percentage change in relative current of the NiO/CC sensitive electrode in a 0.1 M KOH electrolyte containing 1 mM nitrite and 1 mM interfering ions; (**c**) Local magnification of the cyclic voltammograms of five prepared NiO/CC sensitive electrodes in a 0.1 M KOH electrolyte containing 1 mM nitrite; (**d**) Comparison bar chart of peak current decay of five different NiO/CC electrodes in a 0.1 M KOH electrolyte containing 1 mM nitrite; (**e**) Local magnification of the cyclic voltammogram of the NiO/CC sensitive electrode after four stability tests in a 0.1 M KOH electrolyte containing 1 mM nitrite; (**f**) Peak current bar chart of the NiO/CC electrode after storage for 0–4 weeks in a 0.1 M KOH electrolyte containing 1 mM nitrite; (**g**) Local magnification of the cyclic voltammogram of the NiO/CC sensitive electrode after five consecutive tests in a 0.1 M KOH electrolyte containing 1 mM nitrite; (**h**) Comparison bar chart of peak current decay of the same NiO/CC electrode after five consecutive tests in the presence of 1 mM nitrite. All bar graph data (**b**,**d**,**f**,**h**) are presented as mean ± standard deviation (SD, *n* = 3). Error bars represent the standard deviation from three independent replicate experiments. The stability and selectivity claims are supported by statistical analysis rather than visual inspection alone.

In order to systematically evaluate the practical application performance of NiO/CC electrodes in nitrite detection, we conducted comprehensive tests from three dimensions: anti-interference ability, stability, and reproducibility. In the anti-interference experiment, various potential interfering substances with the same concentration were introduced into the 0.1 M KOH solution containing 1 mM nitrite, including inorganic ions (NO_3_^−^, NH_4_^+^, K^+^, Ca^2+^, Zn^2+^, Mg^2+^, Fe^3+^, SO_4_^2−^, CO_3_^2−^, Cl^−^) and organic molecules (urea, glucose) (Figure 6a,b). The cyclic voltammetry test results (Figure 6) showed that the current response changes at the characteristic reduction potential (−0.15 V vs. Hg/HgO) were all less than 3%. This excellent anti-interference ability is mainly attributed to the specific recognition of nitrite by the nickel oxide material and its unique electronic structure, which can effectively inhibit the competitive reduction reactions of interfering substances on the electrode surface.

The NiO/CC electrode demonstrated outstanding long-term stability, retaining 98.11% of its initial current response after four weeks of continuous use (RSD = 1.21%) (Figure 6e,f). This excellent stability can be attributed to the robust structural and chemical properties of the electrode material. Furthermore, the sensor showed high reproducibility, with RSD values of 1.14% for five repeat measurements on a single electrode (Figure 6g,h) and 1.17% for five electrodes prepared independently (Figure 6c,d). These findings confirm the reliability and consistency of the NiO/CC fabrication protocol. In summary, the NiO/CC sensor offers excellent anti-interference, stability, and reproducibility, establishing it as a promising candidate for transitioning from laboratory research to reliable, real-time nitrite monitoring in complex environmental and food samples.

### 3.5. Actual Sample Testing

In order to verify the reliability of the NiO/CC sensitive electrode in practical applications, the nitrite content in pickled vegetables and drinking water samples was analyzed using the standard addition method. Each sample was measured three times to ensure the accuracy of the data. The results are shown in Table 2. In the complex pickled vegetable matrix, the spiked recovery rate of nitrite was between 97.40% and 103.07%, and the relative standard deviation (RSD) was less than 3.37%, indicating that the electrode has good detection accuracy in both simple matrices (drinking water) and complex matrices (pickled vegetables), and can effectively overcome the influence of interfering substances such as organic acids, pigments, and salts. This excellent performance mainly stems from the unique surface chemical properties of nickel oxide and its selective catalytic mechanism for nitrite: The three-dimensional structure formed by nickel oxide nanowires effectively inhibits the interference of macromolecular substances through the sieve effect, and its rich oxygen vacancy active centers on the surface show specific catalytic preference for the reduction reaction of nitrite. The above results fully confirm the reliable application potential of the NiO/CC electrode in environmental water body monitoring and complex food systems, providing key technical support for the development of high-performance electrochemical sensors for practical scenarios.

## 4. Conclusions

This study addressed the limitation of traditional electrochemical nitrite detection methods due to their strong oxidizing properties, which restrict the detection potential to a high level. By using a one-step electrode deposition combined with calcination method, a nickel oxide/carbon cloth (NiO/CC) sensitive electrode rich in oxygen vacancies was successfully prepared. This electrode fully utilized the excellent electrocatalytic reduction property of nickel oxide—its surface oxygen vacancies effectively adsorbed nitrite and promoted the hydrogenation conversion of the key intermediate (*NO) in the reduction reaction, significantly reducing the reaction energy barrier, thereby achieving high sensitivity detection of nitrite at a low working potential of −0.15 V (vs. Hg/HgO). The electrochemical test results showed that the linear detection range of the NiO/CC sensor was from 10 to 500 μM, the detection limit was as low as 0.091 μM (S/N = 3), the sensitivity was as high as 2910 μA·mM^−1^·cm^−2^, and it also exhibited excellent anti-interference ability and stability. This performance advantage fully verified the significant potential of constructing electrochemical sensors based on the catalytic reduction mechanism in reducing the detection potential, providing new research ideas and experimental basis for the rational design of high-performance electrochemical sensing interfaces.

## Figures and Tables

**Table 1:** Comparison of NiO/CC with other nitrite sensors.

Electrode Materials	Detection Method	Sensitivity (µA mM^−1^ cm^−2^)	Linear Range (µM)	LOD (µM)	Detection Potential (V)	Reference
polyNi/glassy carbon electrode	CV	2430	5–542	0.043	+1.0 V vs. Ag/AgCl	[35]
SNPs/CPZ/Nf nanocomposite GCE	DPV	0.0007 μA/μM	20–200	7	+0.12 V vs. Ag/AgCl	[8]
NiCo_2_O_4_ nanotapes	I-t	1030	10–300	1.04	+0.8 V vs. SCE	[36]
AuNPs/MoS_2_/GN	I-t	2900	5–5000	1	+0.8 V vs. Ag/AgCl	[37]
Cunano/CNTs/CS/GCE	I-t	48.92 μA mM^−1^	0.1–2500	0.024	−0.2 V vs. SCE	[38]
Cu/CP	I-t	2140	10–1000	0.079	−0.05 V vs. Hg/HgO	[39]
NiO/CC	CV	2190	200–1000	0.091	−0.15 V vs. Hg/HgO	This work
	I-t	2760	10–100	0.091	−0.15 V vs. Hg/HgO	
		2180	100–500	0.091	−0.15 V vs. Hg/HgO	

**Table 2:** Electrochemical detection of nitrite in real sample.

Actual Samples	Initial (µM)	Added (µM)	Found (µM)	Recovery (%)	RSD (%, *n* = 3)
Drinking water	0	10	10.17	101.70	1.47
		20	21.93	109.65	1.75
		50	51.77	103.54	1.26
sauerkraut	1.17	10	10.88	97.40	2.44
		20	21.82	103.07	3.08
		50	52.52	102.63	3.37

Note: The detected concentrations were calculated using the calibration curve for the low-concentration range (10–100 μM) obtained from chronoamperometric measurements at −0.15 V vs. Hg/HgO. Each sample was measured three times (*n* = 3).

## Data Availability

The data that has been used is confidential.
